# Differential Contributions of Nonmuscle Myosin II Isoforms and Functional Domains to Stress Fiber Mechanics

**DOI:** 10.1038/srep13736

**Published:** 2015-09-04

**Authors:** Ching-Wei Chang, Sanjay Kumar

**Affiliations:** 1 Department of Bioengineering, University of California, Berkeley, Berkeley, CA 94720.

## Abstract

While is widely acknowledged that nonmuscle myosin II (NMMII) enables stress fibers (SFs) to generate traction forces against the extracellular matrix, little is known about how specific NMMII isoforms and functional domains contribute to SF mechanics. Here we combine biophotonic and genetic approaches to address these open questions. First, we suppress the NMMII isoforms MIIA and MIIB and apply femtosecond laser nanosurgery to ablate and investigate the viscoelastic retraction of individual SFs. SF retraction dynamics associated with MIIA and MIIB suppression qualitatively phenocopy our earlier measurements in the setting of Rho kinase (ROCK) and myosin light chain kinase (MLCK) inhibition, respectively. Furthermore, fluorescence imaging and photobleaching recovery reveal that MIIA and MIIB are enriched in and more stably localize to ROCK- and MLCK-controlled central and peripheral SFs, respectively. Additional domain-mapping studies surprisingly reveal that deletion of the head domain speeds SF retraction, which we ascribe to reduced drag from actomyosin crosslinking and frictional losses. We propose a model in which ROCK/MIIA and MLCK/MIIB functionally regulate common pools of SFs, with MIIA crosslinking and motor functions jointly contributing to SF retraction dynamics and cellular traction forces.

Mammalian cells can generate traction forces against solid supports by assembling contractile stress fibers (SFs), which are bundles of filamentous actin (F-actin), actin-binding proteins, and nonmuscle myosin II (NMMII). These traction forces are crucial to a variety of fundamental cellular properties and behaviors, including motility, mechanosensing, shape stability, polarity, and fate determination^1^,^2^,^3^,^4^, and alterations in this force generation have been demonstrated to influence proliferation and apoptosis, stem cell lineage commitment^5^, tissue development, and malignant transformation^6^,^7^,^8^.

NMMII, the key force-generating component in SFs, is a hexamer composed of two regulatory light chains (RLCs), two essential light chains (ELCs), and two heavy chains. The two heavy chains each have a globular head domain containing a binding site for both ATP and actin, a neck domain consisting of sequential IQ motifs that engage one RLC and one ELC. As a molecular motor, NMMII uses ATP hydrolysis to power the rotation of the head domain, which is amplified by the neck domain. Following the neck is the rod domain, containing an α-helical coiled coil, which is responsible for the heavy chain dimerization. Finally, the carboxyl terminal of NMMII features a short non-helical tailpiece^9^,^10^,^11^,^12^. Independent of its motor function, NMMII can also crosslink F-actin into elastic, shear-thickening networks, which has been proposed to contribute to regulation of retrograde flow during migration^13^.

There are three different NMMII isoforms in mammalian cells: NMMII A, B, and C (hereafter MIIA, MIIB, and MIIC). MIIA and MIIB are the predominant NMMII isoforms in many cells^14^ and have been shown to play distinct roles in mediating traction forces during migration. For example, MIIB localizes to the cell rear and is more involved than MIIA in maintaining persistent cell migration, polarization, and durotaxis^15^,^16^,^17^, whereas MIIA localizes to the cell front and preferentially contributes to nascent adhesion formation and cell body translocation^18^,^19^. The function of MIIC is more poorly understood but has been shown to be critical for driving neuronal process outgrowth and regulating cytokinesis in lung and breast cancer cells^20^,^21^,^22^,^23^,^24^. While MIIA has the highest rate of ATP hydrolysis of the three isoforms and therefore propels actin filaments most rapidly, MIIB has a significantly higher duty ratio than MIIA and MIIC and therefore spends the longest time bound to actin in the force-generating state, maintaining tension and crosslinking the SF network^11^,^20^.

MIIA and MIIB both contribute to SF assembly, though the details of these contributions remain incompletely understood. MIIA initiates proto-bundles that can later be stabilized by MIIB, and this process may be involved in the establishment of rearward polarity during migration, which is where MIIB preferentially localizes^18^. However, for spreading cells without particular front-rear polarization, myosin isoform localization to stress fibers may be cell-type-dependent; MIIA is comparatively depleted in central SFs in the human melanoma cell line A-2058^25^ and human MRC-5 fibroblasts^26^, whereas in mouse embryonic fibroblasts (MEFs) MIIB localizes to peripheral bundles where it mediates matrix fiber contraction^27^. Even less is known about how specific functional domains of each isoform contribute to SF mechanics. Past studies have explored contributions of the IQ2 motif (which contains the RLC binding site) and tailpiece (which mediates filament assembly) to MIIA assembly stability in SFs, its recycling to the leading edge, and its localization in SF sub-populations^9^, but have not yet explored domain-specific contributions to SF mechanics. The head but not the IQ2 motif has been shown to be required for driving efficient cytokinesis^28^, implying that these domains differentially drive contractile functions.

In this study, we directly investigate the contributions of specific NMMII isoforms and functional domains to the mechanical properties of single SFs by combining laser nanosurgery, isoform-selective NMMII suppression, and domain rescue studies using NMMII mutants with known mechanochemical defects. This builds on previous work in which we applied laser nanosurgery^29^ to interrogate the mechanics, tension distributions, and structural contributions of different sub-populations of SFs (e.g., central and peripheral) and discovered that they have distinct retraction dynamics, contribute to cell shape stability to different degrees, and are differentially regulated by the myosin activators Rho-associated kinase (ROCK) and myosin light chain kinase (MLCK)^30^,^31^. Our results support a model in which ROCK and MIIA co-regulate the mechanics of central SFs whereas MLCK and MIIB co-regulate the mechanics of peripheral SFs, and in which the motor and crosslinking functions of MIIA control central SF retraction.

## Results

### Expression levels of NMMII isoforms in U373 MG cells

In previous studies, we found U373 MG human glioma cells to be an informative model system for dissecting SF mechanics and compartmentalization^30^,^31^ and thus chose to use this cell line for the current study as well. To design our isoform loss-of-function studies, we first determined the relative abundance of NMMII isoforms in these cells using mass spectrometry (Figure S1), which revealed a molar ratio of MIIA: MIIB: MIIC of approximately 10:5:1. Because of the overwhelming relative abundance of MIIA and MIIB, we focused solely on these two isoforms in subsequent analyses.

### NMMII isoforms: stress fiber viscoelasticity

**Figure 1:**
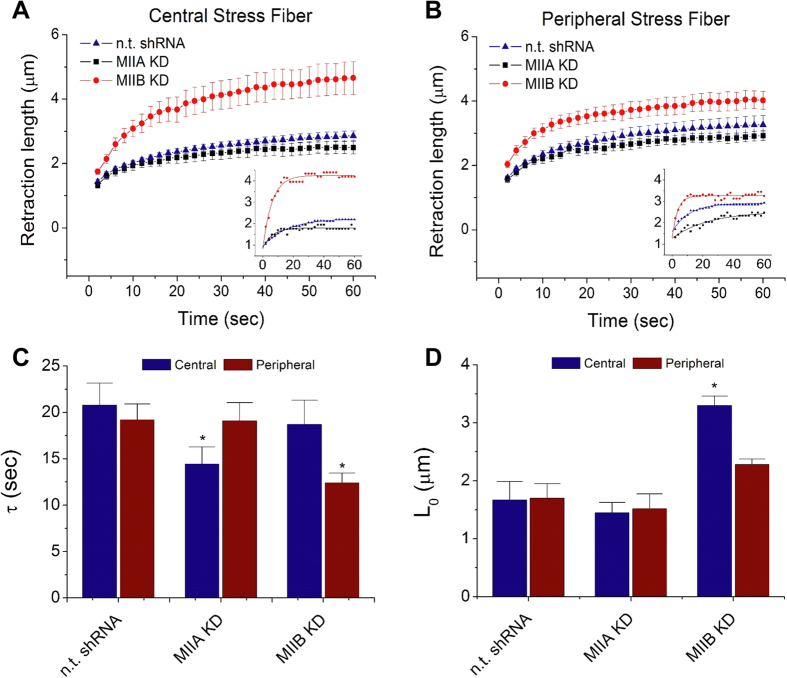
Contributions of NMMII isoforms to SF retraction dynamics. (**A**,**B**) Mean retraction traces of multiple central SFs (**A**) and peripheral SFs (**B**) following transduction with a non-targeting shRNA, or an shRNA directed against MIIA or MIIB. Error bars represent the standard error of the mean (SEM). Insets show representative curves and their fits. Laser ablation was performed immediately after time = 0 and therefore the origin was not included in the fitting model. Instead, a non-zero y-intercept was assigned as one of the fitting parameters. See Methods for the fitting model and detailed description. (**C**,**D**) Viscoelastic time constant (τ) and retraction plateau distance (L_0_) obtained from the Kelvin-Voigt fits. *N* ≥ 13 per condition. Error bars represent SEM. With two-tailed Student’s *t*-tests, statistically significant differences (*p* < 0.05) are denoted by star signs (*) versus the corresponding n. t. (non-targeting) shRNA control.

To investigate the contributions of MIIA and MIIB to SF viscoelastic properties, we performed loss-of-function studies in which we suppressed MIIA and MIIB in U373 MG cells with lentiviral shRNA. Successful isoform-specific suppression was confirmed by immunoblot (Figure S2), with >99% MIIA knockdown (KD), 63% MIIB KD, and no cross-isoform suppression. To confirm that each isoform could still assemble into SFs when the other one was knocked down, we conducted immunofluorescence imaging of MIIA KD or MIIB KD cells along with naïve cells (for direct comparison), which indicated that the other isoform could indeed incorporate into SFs (Figure S3). We then applied femtosecond laser ablation to photodisrupt single SFs in MIIA KD, MIIB KD, or control (non-targeting shRNA-transduced) cells and tracked SF retraction following fiber scission (Figure S4). Following our previous studies, we compartmentalized SFs according to whether they were located at the cell periphery (peripheral SFs) or more centrally (central SFs), based on our finding that these two populations possess different viscoelastic properties and are controlled by different myosin activators^30^,^31^. Retraction curves (retraction distance vs. time; Fig. 1A,B, with insets showing representative curves and fits) reveal that MIIA KD and MIIB KD produce distinct shifts in retraction mechanics and that the nature of these shifts depends on the location of the SF severed. As in our previous studies, we fit each individual retraction curve to a Kelvin-Voigt viscoelastic cable model described by a characteristic time constant (τ), which reflects the fiber’s effective viscosity/elasticity ratio, and a plateau retraction distance (L_o_), which equals the strained SF length before ablation, and performed statistical analysis on the fitted parameters for many SFs (Fig. 1C,D). This analysis revealed that MIIA KD preferentially reduces the retraction time constant for central SFs, whereas MIIB KD reduces the value of that parameter for peripheral SFs (Fig. 1C). MIIA KD did not significantly affect the retraction plateau distance (L_0_) of either SF population, while MIIB KD significantly increased that of central SFs (Fig. 1A,B,D).

### NMMII isoforms: correlations with ROCK and MLCK

From these retraction time constant data, we surmised that MIIA preferentially contributes to the viscoelastic properties of central SFs whereas MIIB does the same for peripheral SFs. Additionally, we noticed that these results qualitatively resembled our previous measurements in which we severed SFs following pharmacologic inhibition of ROCK (Y-27632) and MLCK (ML-7); in these measurements, ROCK and MLCK inhibition preferentially reduced the retraction time constant of central and peripheral SFs, respectively^30^. It therefore occurred to us that ROCK and MIIA (and MLCK and MIIB) may be regulating a common pool of SFs. To more rigorously establish a potential correlation, we performed a two-way analysis of variance (ANOVA, Table S1) against the two data sets. When Y-27632 and ML-7 are grouped together (1^st^ column), their effects on central and peripheral SF (for both τ and L_0_ values) are typically distinct and uncorrelated, indicated by low *p*-values of interaction. The group of MIIA KD and MIIB KD shows similar behavior (2^nd^ column). However, when Y-27632 and MIIA KD are grouped together (3^rd^ column), and ML-7 and MIIB KD are grouped together (4^th^ column), the corresponding *p*-values increase substantially (exhibiting lower or no interactions; comparing the *p*-values in the same color), indicating more strongly correlated effects on SF viscoelastic properties within these groups. In other words, quantitative correlations exist between the effects of ROCK inhibition and MIIA KD on central SF retraction, and between the effects of MLCK inhibition and MIIB KD on peripheral fiber retraction.

**Figure 2:**
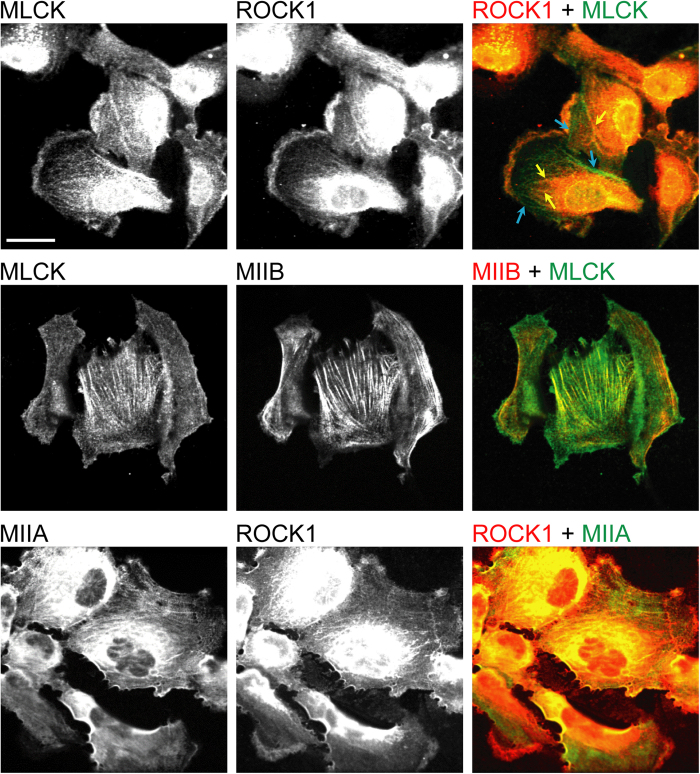
Immunofluorescence confocal optical section images illustrating colocalization of MLCK and MIIB, and of ROCK1 and MIIA. (Top row) Co-staining of ROCK1 and MLCK. Yellow arrows indicate ROCK1-associated central SFs, and light blue arrows indicate MLCK-associated peripheral SFs. (Middle row) Co-staining of MIIB and MLCK. (Bottom row) Co-staining of MIIA and ROCK1. In the top and bottom rows, the image brightness was adjusted to more clearly depict stress fibers in the lamellar regions of the cell, thus necessitating saturation at the cell center. Scale bar: 30 μm.

**Figure 3:**
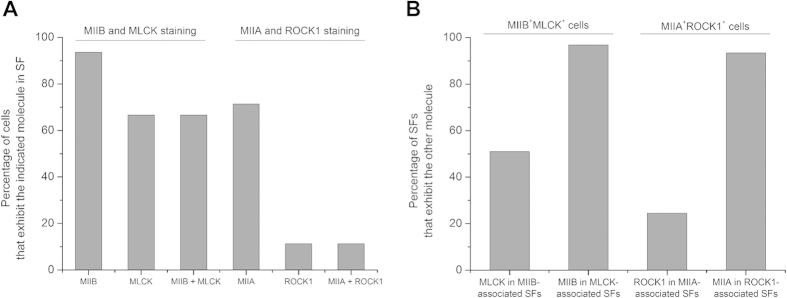
Quantification of immunofluorescence confocal optical section images indicating spatial colocalization between MLCK and MIIB (left halves of each panel), and between ROCK and MIIA (right halves of each panel). (**A**) Percentage of *cells* exhibiting expression of a given protein in its stress fibers. The third (MIIB + MLCK) and sixth (MIIA + ROCK) columns report the percentage of cells with stress fibers containing both listed components. For example, MIIB + MLCK refers to the percentage of cells containing stress fibers that stain positively for both MIIB and MLCK. (**B**) Percentage of myosin-isoform-positive and kinase-positive *stress fibers* that also stained positively for the indicated molecules. All bars in both panels represent data from N > 130 cells taken from multiple biological replicates.

To further substantiate these correlations, we then performed dual immunofluorescence imaging of ROCK1/MLCK, MIIB/MLCK, and MIIA/ROCK1. As reported previously, ROCK1 and MLCK were observed to localize both to stress fibers and to the cytoplasmic background^32^,^33^,^34^,^35^. For cells in which both kinases unambiguously localized to SFs, MLCK preferentially localized to peripheral SFs while ROCK1 localized to central SFs (Fig. 2, top row), consistent with past observations that ROCK and MLCK selectively regulate these two pools of SFs^30^,^33^. More importantly, co-staining of MLCK and MIIB indicated that for cells in which MLCK clearly localized to SFs, the MLCK-positive SFs nearly always (>95%) also stained positively for MIIB (Fig. 2, middle row; Fig. 3, left halves of each panel). The same relationship held for ROCK1 and MIIA (>90% colocalization; Fig. 2, bottom row; Fig. 3, right halves of each panel). These observations further support the notion that MIIB and MLCK are associated with common pools of SFs, as are MIIA and ROCK.

### NMMII isoforms: localization and assembly stability

**Figure 4:**
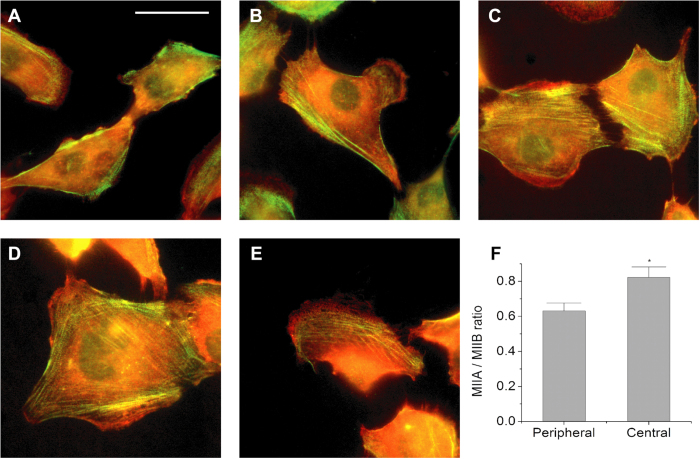
Immunofluorescence confocal optical section images illustrating co-localization of MIIA and MIIB in SFs. (**A**–**E**) Images of MIIB (green) and MIIA (red) in immunostained cells. (**F**) Quantification of the MIIA/MIIB fluorescence intensity ratio in peripheral SF and central SF sub-populations. *N* = 22 cells. Error bars represent SEM. The average ratios are statistically significantly different (*p* < 0.05; two-tailed Student’s *t*-test), indicated by a star sign (*). In all images, the image brightness was adjusted to more clearly depict stress fibers (see Fig. 2 legend). Scale bar: 30 μm. See Methods for the approach to image compartmentalization for different SF sub-populations.

If MIIA and MIIB indeed preferentially contribute to central and peripheral SF contractility, respectively, then one might expect this to be reflected in the regional localization of each isoform. We therefore performed dual-color localization studies in which we immunostained for MIIA and MIIB using isoform-specific antibodies. We readily identified SFs at the cell periphery in which both isoforms localized but were modestly enriched in MIIB (Fig. 4A–E). Where lamellipodia were present, MIIA localized to the lamellipodial edge, consistent with past reports^14^,^18^,^19^,^36^. In our image quantification, we excluded the edges of lamellar areas (where there were no SFs) and defined SFs within 20 pixels (~8.2 μm) from cell edge to be peripheral SFs. The enrichment of MIIB at cell periphery was then verified by quantitative ratiometric analysis (Fig. 4F).

**Figure 5:**
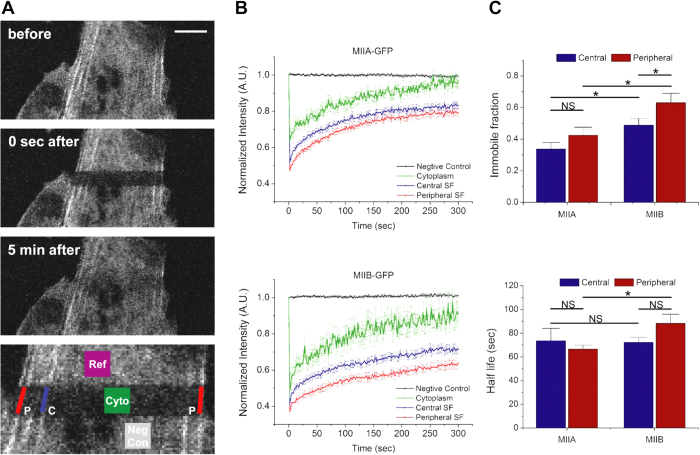
Fluorescence recovery after photobleaching (FRAP) measurements to elucidate NMMII isoform assembly stability in SFs. (**A**) Typical FRAP experiment using MIIB-GFP and confocal optical sectioning. The image brightness was adjusted to more clearly depict stress fibers (see Fig. 2 legend). An image of the enlarged photobleached region is shown (bottom), with ROIs highlighted for two peripheral SFs (red), a central SF (blue), and cytoplasm (green). A reference region in a non-photobleached area was used for the correction of unintentional bleaching (purple). A negative control region (gray), also outside the photobleached area, was selected to confirm successful correction for unintentional photobleaching, following the correction steps using the reference region (see below). (**B**) Fluorescence recovery dynamics of MIIA-GFP (top) and MIIB-GFP (bottom) in central (blue curves) and peripheral (red curves) SF subpopulations as well as in cytoplasm (green curves). The negative controls (black curves) indicate fluorescence dynamics from non-photobleached areas in the corresponding FRAP experiments. (**C**) Quantification of immobile fraction (top) and half-life (bottom) of the fluorescence recovery dynamics. *N* ≥ 50 per condition. Error bars represent SEM. With two-tailed Student’s *t*-tests, statistically significant differences (*p* < 0.05) are denoted by star signs (*). NS: not significant. Scale bar: 15 μm.

While these studies suggest differences in isoform localization, these differences are relatively subtle, which is perhaps not surprising given that MIIA and MIIB can co-assemble within single fibers^37^. This would be consistent with the notion that MIIA and MIIB associate with both populations of SFs, with the stoichiometry dependent upon location. Moreover, these modest localization differences are unlikely to solely explain the differential effects of each isoform on SF mechanics. However, we reasoned that while both isoforms localize with both SF pools, they might do so with different degrees of stability. To explore this possibility, we separately expressed GFP-tagged MIIA and MIIB and performed fluorescence recovery after photobleaching (FRAP) measurements on peripheral and central SFs (Fig. 5A,B). We then fit the recovery curves to a standard first-order model^38^,^39^ to obtain the half-life of recovery and the immobile fraction (i.e., the fraction of fluorescent subunits incapable of exchanging with the bulk). Within each SF population, a larger population of MIIB was immobile, indicating that MIIB more stably associates with both SF populations than MIIA, consistent with previous studies (Fig. 5C, top plot)^17^,^36^. Superimposed upon these differences, we found that a larger fraction of MIIB was immobile within peripheral SFs than within central SFs, consistent with a model in which MIIB associates more stably with peripheral SFs than with central SFs. Recovery half-life results (Fig. 5C, bottom plot) were consistent with this observation; MIIB within peripheral SFs required a longer half life of recovery than MIIA within peripheral SFs, indicating slower or less frequent exchange of MIIB molecules in peripheral SFs and those in the surrounding cytoplasm. This, in turn further implies more stable assembly for the mobile fraction of MIIB at periphery. Together, these data suggest that MIIB more stably associates with peripheral SFs.

### NMMII functional domains: stress fiber viscoelasticity

Having uncovered differential association of MIIA and MIIB with specific SF pools, we next sought to gain deeper insight into the molecular mechanisms through which these isoforms contribute to SF mechanics. Previous studies have shown that the localization of NMMII and its ability to generate force may be attributed to different NMMII functional domains^9^,^28^. We chose to focus our efforts on MIIA, given its twofold enrichment relative to MIIB in these cells (Figure S1) and the fact that the mechanochemistry of MIIA has been significantly more deeply investigated than MIIB. To this end, we performed domain-mapping studies in which we rescued MIIA KD cells with MIIA mutants with known mechanochemical defects.

We rescued MIIA KD cells with four constructs: Wild Type (WT; i.e., full-length protein), ΔHead, ΔIQ2, and ΔTailpiece^9^,^28^. As described earlier, the tailpiece and IQ2 domain play important roles in myosin filament assembly, and their deletion has been shown to influence stress fiber localization to some degree^9^,^28^. However, because all three deletion constructs contain the assembly-competent domain (ACD), which drives myosin filament formation, they may still be incorporated into stress fibers (Figure S5). Importantly, because the head domain is required for actin engagement and the NMMII power stroke, the ΔHead (lacking the head domain) construct is uniquely predicted to lack crosslinking and motor function. For these reasons, comparative study of the ΔHead, ΔIQ2, and ΔTailpiece constructs offers an opportunity to isolate contributions of myosin crosslinking and motor functions to stress fiber mechanics. We note that these cells differ from those considered earlier (Fig. 1) in two important respects: First, these cells express exogenous MIIA; second, SFs in these cells are visualized via an MIIA-based label rather than a Lifeact-based label, which may select for a specific subset of SFs. Thus, the WT rescue represents a key control and the most appropriate comparison for inferring contributions of specific domains to SF retraction mechanics.

**Figure 6:**
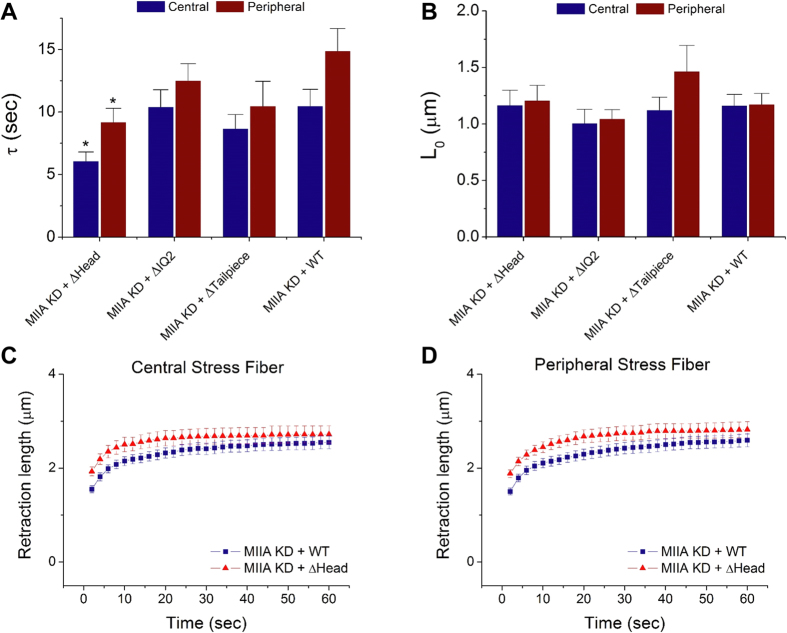
Contributions of specific MIIA domains to retraction parameters. (**A**,**B**) Viscoelastic time constant (τ) and retraction plateau distance (L_0_) obtained from fitting the Kelvin-Voigt model to the SF ablation data for GFP-tagged MIIA constructs (see text for details). *N* ≥ 17 per condition. *p < 0.05 (by two-tailed Student’s *t*-test) relative to the corresponding MIIA KD + WT condition. (**C**,**D**) SF retraction data for central SFs and peripheral SFs under conditions MIIA KD + ΔHead and MIIA KD + WT, which are shown to exhibit distinct viscoelastic time constants in (**A**). Error bars represent SEM.

**Figure 7:**
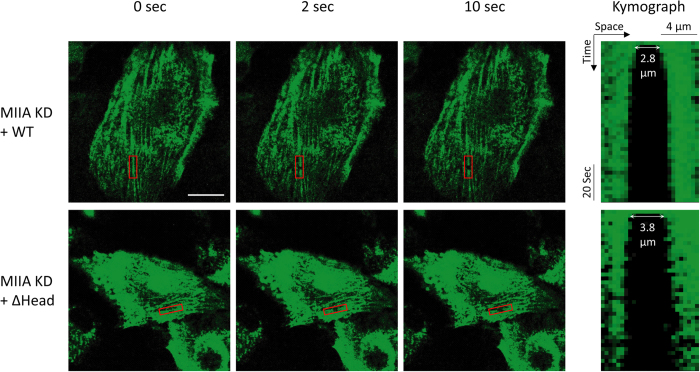
Representative live-cell SF ablation images for MIIA KD + WT and MIIA KD + ΔHead (columns 1–3) and the corresponding kymographs (column 4) illustrating the dynamics of SF retraction highlighted in red boxes. The images were acquired from confocal optical sectioning. The distance between the two severed SF ends (defined as 2L; see Figure S4) at 2 seconds are labeled in the kymographs, which shows that retraction of stress fibers in MIIA KD + ΔHead cells occurs at faster time scales than in MIIA KD + WT cells. For the images and kymograph in the bottom row, brightness was adjusted to more clearly depict stress fibers (see Fig. 2 legend). White scale bar: 20 μm.

**Figure 8:**
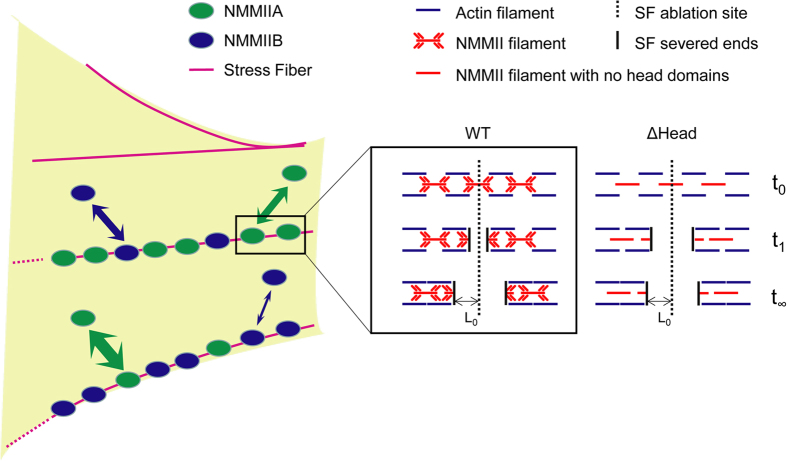
Model describing NMMII isoform localization and stability in SF sub-populations as well as the contribution of NMMII head domain to SF viscoelasticity. (Left) The two-headed arrows represent exchanges of isoforms in SF assembly with those in cytoplasm, with faster exchange reflected by thicker arrows. In this model, we propose that MIIB preferentially contributes to the viscoelastic properties of peripheral SFs due to its greater localization and higher assembly stability in that subset of SFs. Preferential contribution of MIIA to the viscoelastic properties of central SFs can be explained in a similar way. (Right) The contribution of NMMII head domain to SF viscoelasticity is further illustrated in detail. The retraction of a model SF is depicted prior to (t_0_), during (t_1_), and long after (t_∞_) laser incision. Without the NMMII head domain (ΔHead), the severed ends of the SF retract more rapidly than a corresponding SF with the wild-type (WT) NMMII, reflected by the retraction distance at a given time point (t_1_). However, deletion of the head domain does not appear to significantly influence the final retraction distance (L_0_ at t_∞_).

Thus, we transduced U373 MG cells with shRNA to reduce endogenous MIIA background and then rescued with the RNAi-resistant versions of these constructs. To verify that these mutations functionally affected cell-scale force generation, we performed traction force microscopy, in which cellular traction stresses and strain energy per unit area are measured based on the motions of fiduciary particles embedded in a compliant hydrogel substrate (Figure S6)^29^,^31^,^40^,^41^. None of the constructs gave rise to traction force and strain energy/area levels that were statistically distinguishable from the MIIA KD, with the exception of full length MIIA, which rescued traction forces and strain energy/area to roughly 80% and 40% of the values for naïve cells, respectively. We then performed SF photo-disruption experiments to study the contributions of these constructs to SF mechanics (Fig. 6). While the SF retraction plateau (L_0_) with the constructs did not appear to differ from the WT rescue (Fig. 6B), we were surprised to observe that deletion of the head domain reduced the time constant of SF retraction relative to rescue with full length MIIA (Fig. 6A,C,D; Fig. 7). This is consistent with a scenario in which the effective viscosity or internal friction of the stress fiber decreases in the absence of the head domain (∆Head) (Fig. 8). In this case, the retraction is presumably driven by MIIB and is associated with compromised whole-cell traction force.

## Discussion

In this study, we investigated the contributions of NMMII isoforms and MIIA mechanochemical domains to the viscoelastic retraction of individual SFs. SF viscoelastic retraction following MIIA and MIIB suppression qualitatively phenocopied the effects of ROCK and MLCK inhibition, respectively. The notion that MIIA/ROCK and MIIB/MLCK may regulate common pools of SFs is further supported by dual immunostaining studies, which reveal co-localization. Moreover, subcellular localization and FRAP support the notion that MIIB associates more abundantly and stably with peripheral SFs. To gain additional mechanistic insight into the roles of specific MIIA domains in governing SF retraction, we performed rescue studies with domain fragments with known mechanochemical defects. These studies revealed that rescue of MIIA-suppressed cells with a headless MIIA fragment surprisingly speeds viscoelastic retraction relative to the full length rescue (Fig. 8). While the increase in retraction speed with myosin suppression appears somewhat counterintuitive, it is important to remember that the time constant represents the relative contributions of viscosity and elasticity to retraction. Our observations are therefore consistent with a framework in which actomyosin crosslinking functions exert viscous drag on SF retraction, such that disruption of these crosslinks speeds retraction even in the absence of motor function (we explore this idea in greater detail below). To our knowledge, these represent the first measurements of myosin contributions to SF contractile mechanics at the single-fiber level.

Our isoform-specific analysis builds on a wealth of recent studies that have explored subcellular compartmentalization of MIIA and MIIB, especially in the context of cell migration, and have sought to relate mechanochemical differences between isoforms with cellular function. For example, a number of elegant studies have established that NMMII isoforms are highly polarized during two-dimensional, lamellipodium-driven cell migration, with MIIA exclusively populating regions of dynamic protrusion, including the lamellar and lamellipodial regions, where MIIA presumably serves to drive retrograde flow^14^,^18^,^19^,^36^. By contrast, MIIA and MIIB colocalize and cooperate in regions of static, highly contractile bundle formation, with MIIA specifically localizing to the extreme cell rear to promote trailing-edge retraction^11^. This is consistent with the motor function of each isoform, in that MIIA cycles ADP/ATP much more rapidly than MIIB whereas MIIB has a much higher duty cycle than MIIA^10^,^11^,^20^. For these reasons, MIIB is regarded as key to generation of sustained loads^11^. These localization patterns are consistent with our results, in that we observe MIIA in lamellipodia (Fig. 4), as well as substantial overlap in localization patterns across SF populations. The transition from MIIA-rich central SFs to MIIB-rich peripheral SFs would suggest that MIIA and MIIB are dynamically recruited as SFs evolve. Placing this in more standard SF taxonomy^42^, as SFs evolve from centrally-located transverse arcs and immature ventral SFs to peripherally-located and more mature ventral SFs, they become increasingly dominated by MLCK/MIIB-based contractility, which lends itself to the sustained force generation needed to stabilize rear adhesions prior to tail retraction (Fig. 4).

While the field’s understanding of NMMII isoform localization has begun to come into focus, its analogous understanding of the relationship between NMMII activators and isoforms remains incomplete. Our data suggest that SFs may be broadly classified into ROCK/MIIA- and MLCK/MIIB-based populations. Previously, ROCK inhibition has been shown to differentially influence the solubility of MIIA and MIIB, with ROCK inhibition distributing MIIA from bundles to cytoplasm more than MIIB^43^,^44^. However it is important to note that the evidence for these connections is still largely correlative. Additional data are still needed to more definitively validate these regulatory connections, especially a direct interaction between effector and isoform. For example, ROCK/MLCK inhibition studies followed by immunoprecipitation of MIIA and MIIB with their associated RLCs still attached^45^ may help determine whether individual isoform activity is regulated directly by a specific kinase.

To further elucidate the domain-specific contributions of MIIA to SF mechanics, we conducted SF photo-disruption and traction force microscopy studies after rescuing MIIA-suppressed cells with MIIA mutants with known mechanochemical defects. Our results demonstrate that deletion of the head domain speeds the retraction of both central and peripheral SFs, but does not significantly alter SF retraction plateau distance (Figs 6, 7, 8). As discussed earlier, the time constant of retraction reflects the relative influences of viscous drag and SF elastic recoil during retraction. It may initially seem surprising that reduction of actomyosin crosslinking and motor activity within the SF, a perturbation expected to retard sarcomere compaction, would speed retraction. However, the classical Kelvin-Voigt model on which this interpretation is based does not explicitly incorporate the effect of myosin motors, which manifest themselves in both elastic recoil and frictional losses. Indeed, when SFs are modeled as a series of compound Kelvin-Voigt/motor units^46^ the viscoelastic time constant decomposes into two terms: One associated with the passive viscoelastic properties of the SF and another associated with frictional losses within the motor, which is proportional to the stall force of the motor. Deletion of motor function would dramatically reduce the stall force and therefore be expected to reduce the latter term. In addition, actomyosin crosslinking may provide a resistance to filament sliding and retard SF retraction, such that removal of the myosin head domain would eliminate this crosslinking and free the severed ends to retract more rapidly. Interestingly, these changes in the retraction time constant were not accompanied by changes in the plateau retraction distance, whereas a combined Kelvin-Voigt/motor model would predict that the contraction length would also fall with decreasing stall force. However, this model describes a *local* contraction length, whereas our measurements measure the contraction of the entire SF. If the SF is pinned along its length to other cytoskeletal elements or the underlying matrix, as one might expect^46^,^47^,^48^, these adhesive points would be expected to dictate the maximal retraction distance of the SF and thus mask differences in local contraction. We anticipate that SF retraction measurements with adhesive patterns of defined geometry and spacing may help to clarify this issue.

Notably, introduction of either the ∆IQ2 or ∆Tailpiece construct reduces traction forces without altering SF viscoelastic properties. This is somewhat surprising given that deletion of the IQ2 domain has been shown to render MIIA constitutively active in *Dictyostelium*^49^. That said, reintroduction of ∆IQ2 to MIIB-suppressed COS-7 cells does not fully rescue cytokinesis^28^, implying impaired motor function and potentially reflecting differences between *Dictyostelium* and mammalian myosins. The divergence between the traction force and retraction measurements is consistent with a model in which removal of the IQ2 motif or the tailpiece suppresses MIIA motor functions without substantially compromising MIIA crosslinking functions^28^,^50^. This result may also originate in part from a potential role played by these domains in mediating the assembly and subcellular localization of MIIA to specific pools of SFs^9^,^50^. Additional rescue studies with precisely targeted mutations together with high-resolution ultrastructural imaging may help clarify this issue.

In summary, our study lends insight into the molecular biophysical basis of cell migration and contractility by exploring the role of specific NMMII isoforms in the mechanics and dynamics of specific SF compartments. By combining single-SF nanosurgery studies with gain- and loss-of-function studies, our work also illustrates the importance of crosslinking and motor functions mediated by the MIIA head to SF retraction dynamics and cellular traction.

## Methods

### Cell culture

U373 MG human glioblastoma cells stably transduced to express mCherry-Lifeact^30^,^31^ were plated on 35 mm No. 1.5 coverslip-bottomed dishes (thickness = 0.16–0.19 mm; MatTek Corporation) coated with fibronectin at a density of 10 μg/cm^2^, and then cultured at 37 °C supplied with 5% CO_2_ until imaging. Cultures were imaged at low (~40%) confluence to minimize cell–cell contacts. For experiments involving transient transfection with GFP-tagged MIIA constructs, naïve cells not expressing mCherry-Lifeact were used. The omission of the mCherry-Lifeact allowed the use of mCherry shRNA selection markers and was rendered dispensable by the fact that the GFP-tagged MIIA constructs provided adequate visualization of SFs. As a note on cell source, we obtained our U373 MG cells from the UC Berkeley Tissue Culture Facility, which originally obtained these cells from the American Type Culture Collection (ATCC). These cells are now widely acknowledged to share a common origin with U251 MG cells, another human glioblastoma line^51^.

### Non-muscle myosin IIA and IIB suppression and rescue studies

MIIA and MIIB were suppressed via shRNA lentiviral constructs^52^, which were stably transduced into mCherry-Lifeact-expressing U373 MG human glioma cells. More specifically, cells were stably transduced with GFP-MIIA-shRNA, GFP-MIIB-shRNA, GFP-non-targeting-shRNA (negative control), or only GFP, at a multiplicity of infection (MOI) of 10. All transduced cells were sorted for medium to high GFP fluorescence, according to a previously reported protocol^52^. For rescue studies, an mCherry version of MIIA-shRNA was used instead of the GFP-tagged version in naïve U373 MG cells not expressing mCherry-Lifeact, followed by transient transfection with GFP-tagged MIIA ΔHead, ΔIQ2, ΔTailpiece, or WT^9^,^28^ using Lipofectamine LTX (Life Technologies) following manufacturer protocols.

### Immunoblot and immunofluorescence

Immunoblot and immunofluorescence studies were performed as previously described^40^. Both of them employed anti-MIIA (Covance, for immunoblot; Abcam, for immunofluorescence) and anti-MIIB (Covance, for both immunoblot and immunofluorescence) antibodies. In immunofluorescence studies, Alexa 647–phalloidin (Life Technologies), MLCK antibody (Sigma-Aldrich), and ROCK1 antibody (Millipore) were also used.

### Laser nanosurgery and SF retraction

All laser nanosurgery experiments were performed based on previously published protocols^29^,^30^,^31^, using a Zeiss LSM 510 AxioImager 2-photon & confocal microscope. Briefly, confocal images of mCherry or GFP were recorded with 543 nm or 488 nm excitation, respectively, and the selected SF was ablated using a mode-locked MaiTai Ti:saphire femtosecond laser (Spectra Physics) at 770 nm (Figure S4A).

SF retraction distance was recorded every 2 seconds for 80 seconds following ablation. The retraction dynamics was fit to a Kelvin-Voigt model^29^,^30^ (Figure S4B):


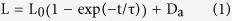


where L, as a function of time, t, is the retraction distance, defined as half the distance between the two severed ends, D_a_ is the length of SF destroyed by the ablation event, L_0_ is the retraction plateau distance, and τ is the viscoelastic time constant. Incised SFs that had not reached a plateau (defined as retraction rate >0.01 μm/s) by 80 sec were deemed to have begun to depolymerize and were excluded from further analysis. In terms of the physical interpretation of the fitting parameters, τ is the ratio of viscosity to elasticity, whereas L_0_ is proportional to the ratio of pre-stress to elasticity. While elasticity plays a role in both parameters, the impact of viscosity and pre-stress can be separated by using this model.

### Myosin localization analysis by SF image compartmentalization

Central and peripheral SF compartmentalization was performed on phalloidin images using MATLAB (MathWorks). Cell area was first outlined manually and lamellipodial regions exhibiting no SFs, if any, were excluded. This image, showing only the cell of interest, was used as the input image. Background, defined by the input image after two-dimensional moving averaging (size = 10 pixels), was subtracted from the input image itself. The SF binary mask was then generated from the background-subtracted image using an intensity threshold of 2% of the maximum intensity of the same image. To define cell periphery, another binary mask was generated from the input image using an intensity threshold of 15% of the maximum intensity of the background-subtracted image, followed by morphological opening and closing with a disk-shaped structuring element of radius = 6 pixels. The resulting image would form a binary mask of cell area, and cell periphery could be defined as the pixels with a local positive divergence of gradient. These cell peripheral pixels were then inflated by convolution with a 40 pixel × 40 pixel square to generate a mask that was further superimposed onto the SF binary mask to generate a peripheral SF binary mask. The SF pixels outside the peripheral SF binary mask then formed a central SF binary mask. These compartmentalized SF masks were then applied to the MIIA/MIIB ratio image for quantitative localization analysis.

### Fluorescence recovery after photobleaching

U373 MG human glioblastoma cells transiently transfected with either MIIA-GFP or MIIB-GFP were employed and fluorescence recovery after photobleaching was conducted using a Zeiss LSM 510 AxioImager confocal microscope. A rectangular region of the GFP channel image was photobleached using a 488 nm laser with 100 iterations and the recovery of fluorescence was recorded every 2 seconds until 300 seconds after photobleaching. During image processing, regions of SFs (excluding the neighboring cytoplasmic area) were selected for quantitative analysis. Cytoplasmic areas were also selected in separate regions for comparisons. To correct for undesired photobleaching (photobleaching that occurred during the 300-second recording following the intentional photobleaching), a large-size region outside the intentionally photobleached area was selected as a reference region. Based on the average intensity of this reference region in the corresponding frame relative to the first frame, the intensities of all the pixels in every frame were normalized to the first frame. Another small region outside the intentionally photobleached area was selected as a negative control to confirm that the undesired photobleaching had been corrected for (by showing no significant intensity decrease in the 300-second time course after correction).

### Traction force microscopy

Traction force microscopy was performed as previously described^40^,^41^ using fibronectin-coated 4 kPa polyacrylamide gels embedded with blue-green beads (Cat. No. F13080, Life Technologies). Maps were generated using Fourier transform traction cytometry^53^.

## Additional Information

**How to cite this article**: Chang, C.-W. and Kumar, S. Differential Contributions of Nonmuscle Myosin II Isoforms and Functional Domains to Stress Fiber Mechanics. *Sci. Rep.* **5**, 13736; doi: 10.1038/srep13736 (2015).

## Supplementary Material

Supplementary Information
