# Supplementary material for: Differential Contributions of Nonmuscle Myosin II Isoforms and Functional Domains to Stress Fiber Mechanics
Source: Sci Rep. 2015 Sep 4;5:13736. doi: 10.1038/srep13736 (PMC4559901; doi:10.1038/srep13736)
Supplement: Supplementary Information [file srep13736-s1.pdf]

Supplementary Information

Differential Contributions of Nonmuscle Myosin II Isoforms and Functional Domains to Stress Fiber Mechanics

Ching-Wei Chang and Sanjay Kumar

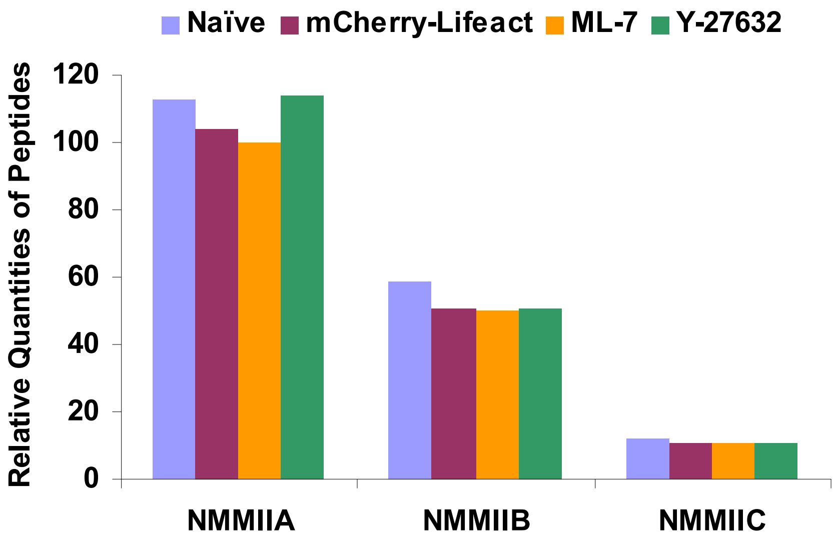

Figure S1. Relative quantities of the NMMII isoform peptides in U373 MG cells, measured by mass spectrometry. Naïve: cells without mCherry-Lifeact transduction and drug treatments; mCherry-Lifeact: cells transduced with mCherry-Lifeact but not treated with any drugs; ML-7: cells transduced with mCherry-Lifeact and treated with 10 μM ML-7 for one hour; Y-27632: cells transduced with mCherry-Lifeact and treated with 5 μM Y-27632 for one hour. As expected, the viral transduction of mCherry-Lifeact for SF labeling did not influence the isoform levels and the drug treatments also had little impact on the isoform levels on a short-time scale.

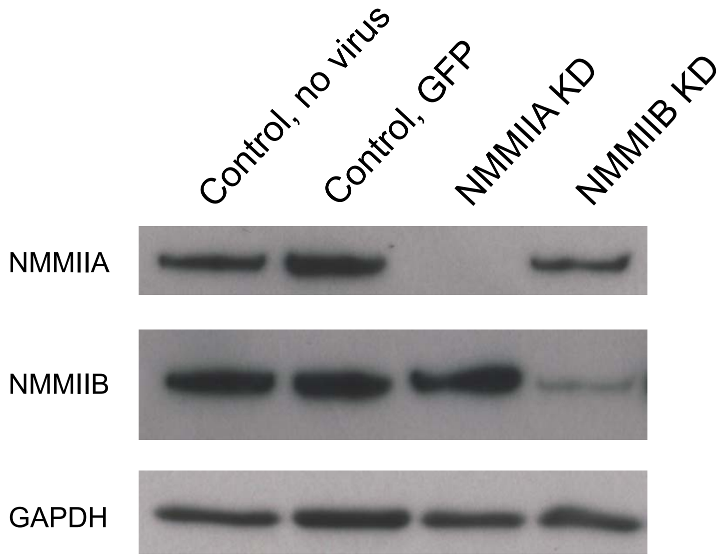

Figure S2. Immunoblot analysis of NMMII isoform knockdown (KD) U373 MG cells along with non-viral-infected and GFP-only infected controls. All infected cells received viral particle treatment at MOI = 10. Quantification of the images revealed >99% NMMIIA suppression and 63% NMMIIB suppression relative to GFP-only control cells.

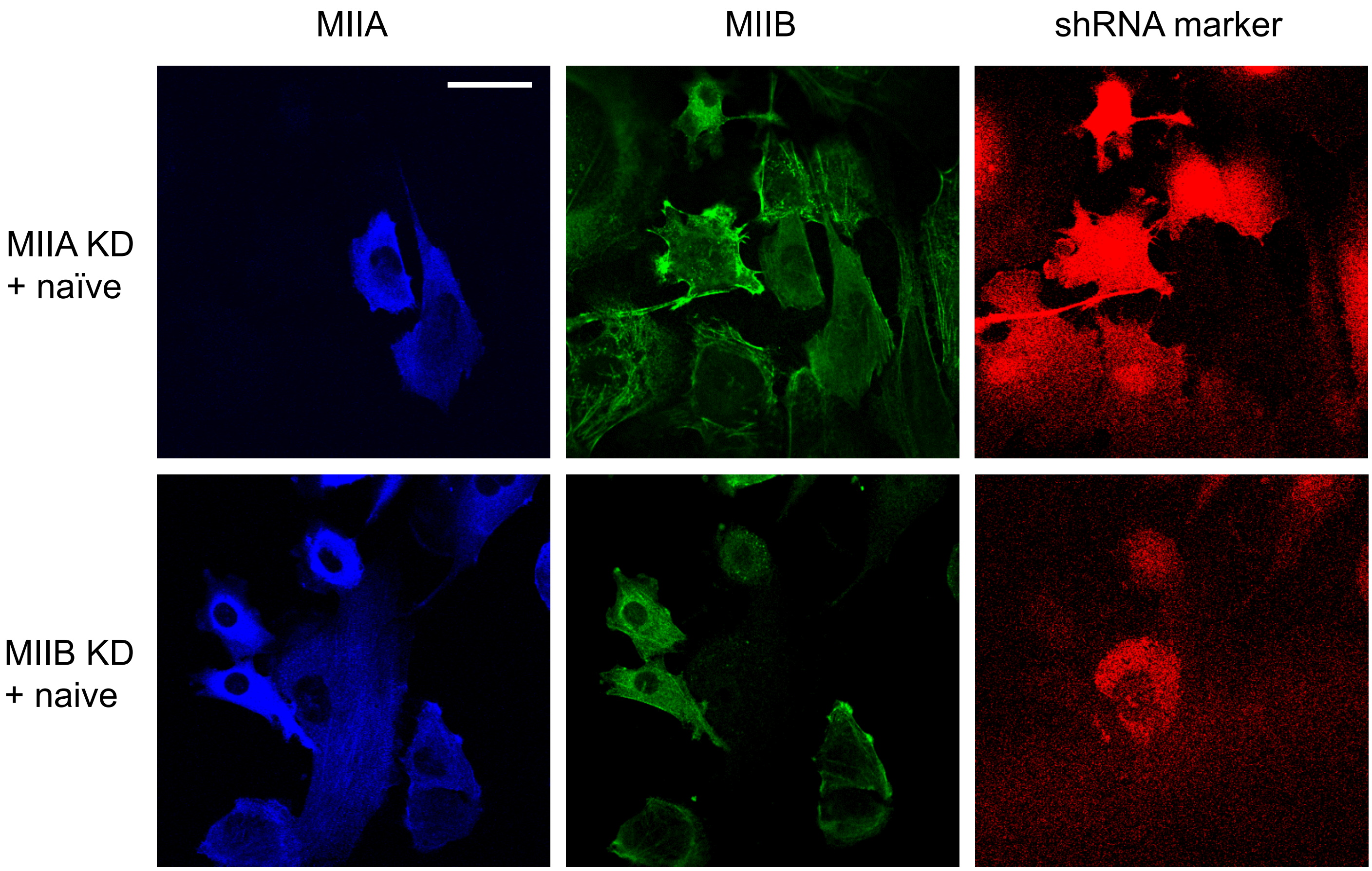

Figure S3. Immunofluorescence confocal optical section images revealing that MIIA and MIIB can still constitute SFs when the other is absent. Images of MIIA KD cells co-cultured with naïve cells (top row) indicate that in cells where shRNA was present (red cells in the right panel), no MIIA was expressed (left panel) while MIIB was still able to contribute to SF structure (middle panel). Similarly, images of MIIB KD cells co-cultured with naïve cells (bottom row) indicate that in cells where shRNA was present (red cells in the right panel), low level of MIIB was expressed (middle panel) while MIIA was still able to contribute to SF structure (left panel). In the first and second columns, the image brightness was adjusted to more clearly depict stress fibers (see Fig. 2 legend). Scale bar: 30 μm.

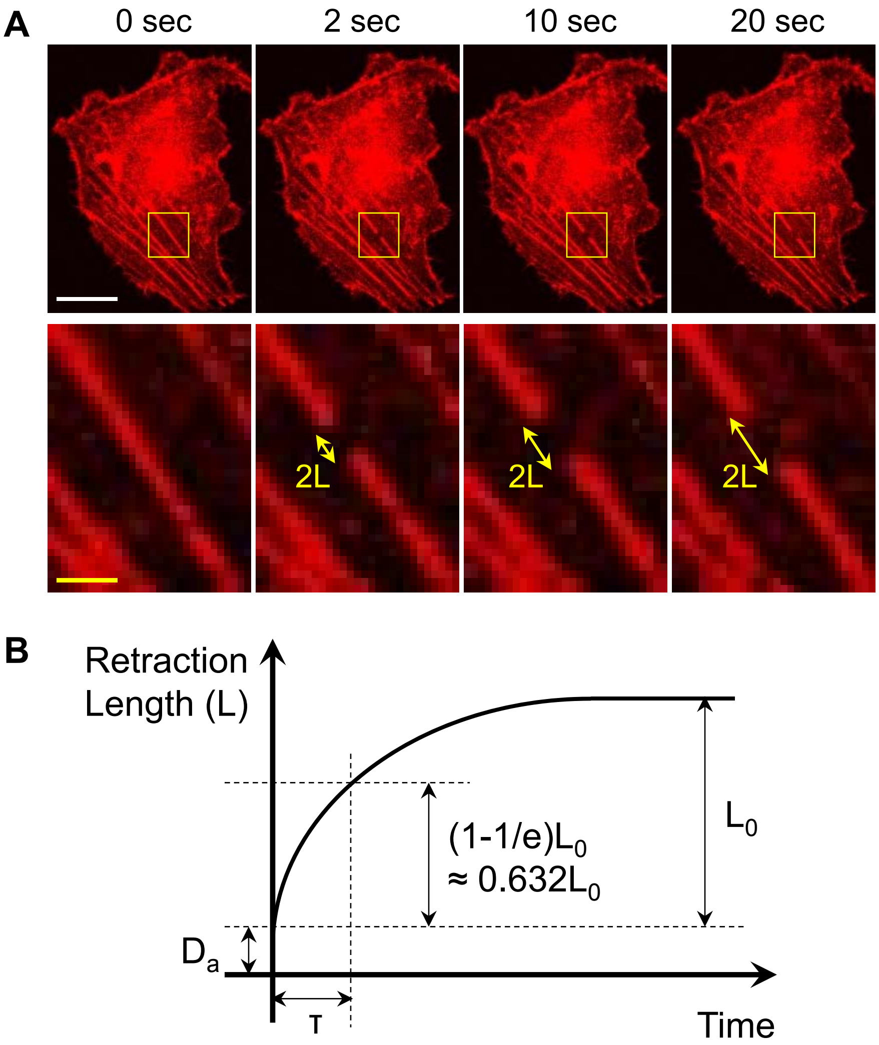

Figure S4. Time-lapse imaging of stress fiber ablation and analysis of retraction. (A) mCherry-Lifeact fluorescence images of SF ablation, acquired from confocal optical sectioning (top row), with boxed region shown at high magnification (bottom row). The retraction length (L) at each time point is defined as half the distance between the two severed ends. White scale bar: 20 μm. Yellow scale bar: 3 μm. (B) Extraction of parameters from curve fits. Data points are fit using a Kelvin-Voigt model with fitting parameter D_a (the length of SF destroyed by the ablation event), τ (viscoelastic time constant), and L_0 (retraction plateau). Brightness of all images was adjusted to more clearly depict stress fibers (see Fig. 2 legend). See Methods for the fitting model and detailed description.

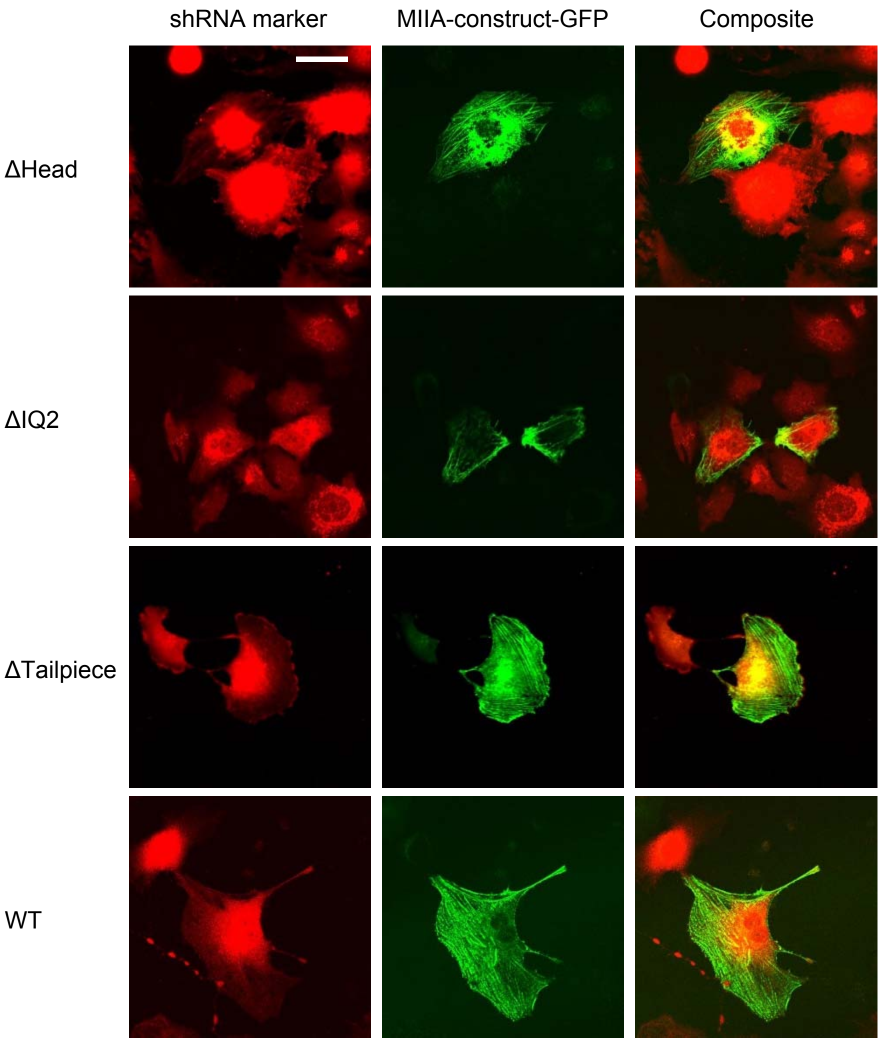

Figure S5. Live-cell confocal optical section images demonstrating that all four siRNA-resistant MIIA constructs (ΔHead, ΔIQ2, ΔTailpiece, and WT in the first, second, third, and fourth rows, respectively) localized to SF structures (second column) in MIIA KD cells (first column). Brightness of the green channel was adjusted to more clearly depict stress fibers (see Fig. 2 legend). Scale bar: 30 μm.

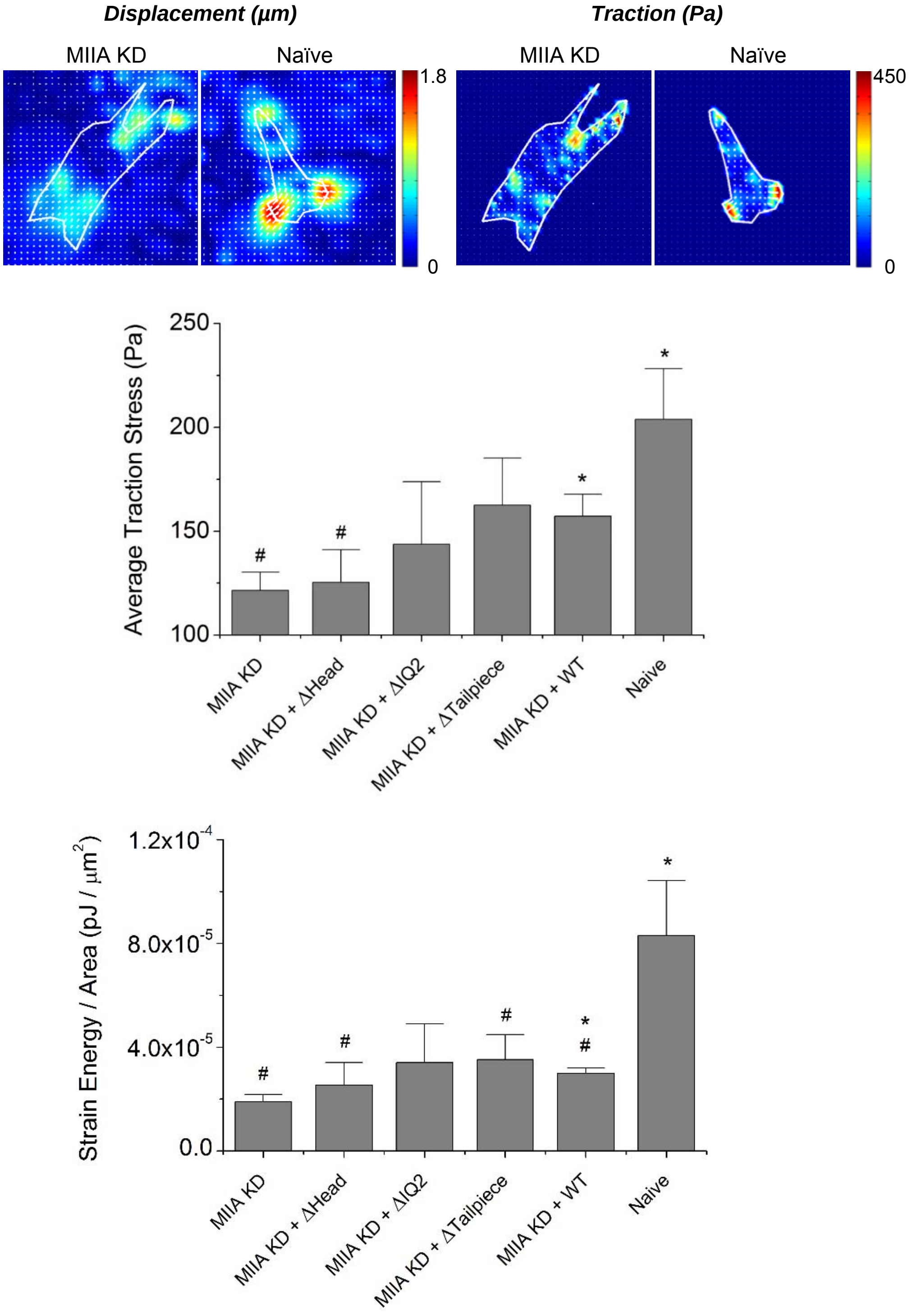

Figure S6. Traction and the corresponding strain energy per unit area generated by MIIA KD cells, various MIIA KD + construct cells, and naïve cells on polyacrylamide gels with stiffness of 4 kPa (middle and bottom, respectively). Representative bead displacement and traction maps of MIIA KD and naïve cells are also shown (top). $N \geq 8$ per condition. Error bars represent SEM. Statistically significant differences ($p < 0.05$ from two-tailed Student's t-tests) are denoted by pound signs (#) compared with the "naïve" condition and by star signs (*) compared with the "MIIA KD" condition.

Table S1. Two-way analysis of variance (ANOVA) of the effect of NMMII isoform KD and drug treatments [1] on the viscoelastic time constant (τ) and the retraction plateau distance (L_0), following central SF (CSF) and peripheral SF (PSF) ablation. The *p*-values are derived from the τ and L_0 values in Fig. 1C, D and the corresponding values with drug treatments in the reference[1].

	Interaction p-value			
	Y-27632 ML-7	***MIIA KD MIIB KD***	***Y-27632 MIIA KD***	***ML-7 MIIB KD***
CSF τ / PSF τ	***p = 0.00021***	***p = 0.0041***	***p = 0.11***	***p = 0.037***
CSF L_0 / PSF L_0	***p = 0.24***	***p = 0.025***	***p = 0.63***	***p = 0.61***

References

1 Tanner, K., Boudreau, A., Bissell, M. J. & Kumar, S. Dissecting regional variations in stress fiber mechanics in living cells with laser nanosurgery. *Biophys J* **99**, 2775-2783 (2010).
